# Brain Activity and Tinnitus

**DOI:** 10.1371/journal.pmed.0020194

**Published:** 2005-06-28

Exposure to short periods of very loud noise can cause tinnitus—a persistent ringing or buzzing in the ears that cannot be blocked out. Tinnitus may affect around 10%–15% of the population; severe tinnitus is very debilitating (1%–2% of the population). Previous work has shown that tinnitus has a neurophysiological basis, but precisely which parts of the brain and the auditory circuits are involved is not yet understood.

The human ear is essentially a very sensitive vibration sensor, one that is able to receive the minute longitudinal vibrations in air that make up sound waves. It can detect sounds from 20 Hertz (Hz) (very low pitch) to 20,000 Hz (very high pitch) but is particularly sensitive to sounds in the range of 500–5,000 Hz—the so-called speech frequencies. However, the ear, and in particular the cochlea, or inner ear, can be damaged by exposure to excess noise, leading to permanent damage to the ear, i.e., deafness.

Some studies in both animals and humans have suggested that tinnitus and hearing loss may be related. These studies have found that neurons in regions of the auditory cortex that have been deprived of stimuli because of hearing loss change their receptive field and may develop enhanced spontaneous activity. Other studies, such as some involving neuroimaging using positron emission tomography, have suggested that parts of the brain involved in attention and emotional regulation might be involved in the production of tinnitus.

One of the key research targets in tinnitus has been investigation of cortical activity, especially in animal models of tinnitus, but studies in humans have been rare. Previous studies have identified temporal and frontal temporal changes in individuals whose tinnitus is severely disabling; however, there have been no group studies comparing abnormalities of ongoing, spontaneous neuronal activity in people with and without tinnitus.

In this month's *PLoS Medicine*, Nathan Weisz and colleagues studied 17 patients with chronic tinnitus and hearing loss and 16 control individuals with normal hearing. Patients were asked to fill in a questionnaire about the impact of tinnitus on their lives and had their levels of tinnitus assessed.

The team's methods differed from previous work in that the team chose to examine the power spectrum of neuromagnetic oscillatory activity during rest, whereas previous studies had focused on measuring neurophysiological responses following sounds.

Normally in awake and healthy subjects a certain rhythm of brain activity at 8–12 Hz—the so-called alpha rhythm—is dominant. Finding enhanced slow-wave, or delta, activity (<4 Hz) in awake subjects is usually a sign of a dysfunctional neuronal network, as these waves can be observed in various neurological and psychiatric disorders. Weisz and colleagues' analysis of the frequency spectrum of recorded magnetic fields revealed that the energy in the alpha band was strongly reduced and that of the delta band enhanced in the group with tinnitus compared with the individuals with normal hearing. This pattern was particularly pronounced in the temporal regions, and overall the effects were stronger for the alpha than for the delta frequency band.

This is the first study to show these changes in delta and alpha spontaneous cortical activity, say the authors. But they concede it is still unclear whether the enhancement of delta activity compared with alpha is the abnormal activity perceived as tinnitus. However, the fact that regions that show slow-wave activity during slow-wave sleep are also regions of low alpha activity supports the idea that changes in cortical activity might be mediated by sensory deprivation, in this case that partial hearing loss might be involved in producing tinnitus.

Tinnitus-related distress as assessed by the questionnaire was strongly associated with this abnormal spontaneous activity, especially in the right temporal and left frontal areas, thus pinpointing a possible tinnitus-related cortical network.

A limitation of this study was that the tinnitus group also had high-frequency hearing loss, whereas the control group did not; the ideal control group would have been patients with the same sort of hearing loss but no tinnitus.

In discussing their findings, the authors suggest that their study supports previous work indicating that the prefrontal cortex is a candidate region for integration of the sensory and emotional aspects of tinnitus. Further studies should focus on frontal areas, which could allow identification of interactions and modulating influences that higher-order psychological processes (e.g., emotions and thoughts) may have on the generation of tinnitus in the auditory cortex.

